# CISAPS: Complex Informational Spectrum for the Analysis of Protein Sequences

**DOI:** 10.1155/2015/909765

**Published:** 2015-01-06

**Authors:** Charalambos Chrysostomou, Huseyin Seker, Nizamettin Aydin

**Affiliations:** ^1^Department of Genetics, University of Leicester, University Road, Leicester LE1 7RH, UK; ^2^Department of Computer Science and Digital Technologies, Faculty of Engineering and Environment, The University of Northumbria at Newcastle, Newcastle-upon-Tyne NE1 8ST, UK; ^3^Department of Computer Engineering, Yildiz Technical University, 34220 Istanbul, Turkey

## Abstract

Complex informational spectrum analysis for protein sequences (CISAPS) and its web-based server are developed and presented. As recent studies show, only the use of the absolute spectrum in the analysis of protein sequences using the informational spectrum analysis is proven to be insufficient. Therefore, CISAPS is developed to consider and provide results in three forms including absolute, real, and imaginary spectrum. Biologically related features to the analysis of influenza A subtypes as presented as a case study in this study can also appear individually either in the real or imaginary spectrum. As the results presented, protein classes can present similarities or differences according to the features extracted from CISAPS web server. These associations are probable to be related with the protein feature that the specific amino acid index represents. In addition, various technical issues such as zero-padding and windowing that may affect the analysis are also addressed. CISAPS uses an expanded list of 611 unique amino acid indices where each one represents a different property to perform the analysis. This web-based server enables researchers with little knowledge of signal processing methods to apply and include complex informational spectrum analysis to their work.

## 1. Introduction

If it is considered that a protein's biological function is controlled by a selective ability of the protein to interact with selected elements in the environment, the following argument arises: how is this selective ability achieved? Several attempts have been made to decode such characteristic features that help drive biological functions of the proteins directly from primary structure of a protein sequence. One common method used for analysing protein sequences to determine biological functions is based on the search for similarities in the arrangements between the groups of sequences. One example is the basic local alignment search tool (BLAST) [1]. Another method for analysing macromodule sequences is to extract structural and physicochemical features, such as amino acid composition and dipeptide composition derived from the primary structure of a protein sequence. These features can be used for various purposes that include prediction of protein structural classes [2, 3], functional classes [4, 5], and protein-protein interactions [6, 7].

In recent years, signal processing techniques have been used in bioinformatics to extract information that is expected to reveal protein's biological function [8–11]. One of the methods that use discrete Fourier transform (DFT) is informational spectrum analysis (ISA) [12, 13]. In previous applications where ISA was used for each group of proteins analysed [12, 13] there was a group of proteins that correspond to specific peaks in the frequency spectrum. Every biological function corresponds to one unique or a set of unique peaks. The importance of this general conclusion is that specific biological functions can be extracted from protein sequences using signal processing techniques by identifying significant features of the frequencies which are not found in unrelated frequencies. However, complementary information such as real and imaginary frequency spectra can be derived from DFT which has successfully been used in various areas including biomedicine [14] but was not previously explored in the analysis of protein sequences. A new method, the complex informational spectrum [15], was proposed and developed, which considers all three frequency spectra for analysing protein sequences, in order to identify new and complementary information in relation to functional properties of the proteins under investigation.

In the traditional approach, due to the complex nature of proteins and their functional groups, the use of only the absolute spectrum in the analysis of protein sequences using the informational spectrum analysis is insufficient, as biologically related features to the analysis of protein sequences can be more distinct either in the real or the imaginary spectrum. Various applications, such as development of new drugs [16], identification of important protein sequence's domains [17], and investigation of protein sequences interaction [18], where ISA and resonant recognition model (RRM) [19] are already applied in the literature, and complex informational spectrum analysis (CISA) [15] will also be applicable and will be able to contribute additional information.

To be able to proceed with current signal processing techniques, a set of numerical values must be assigned to nucleotides or amino acids [20]. These values should represent natural biological characteristics of the macromodules with which they are paired and be relevant to the biological activity of each module. These values can be any of the biochemical properties such as electron-ion interaction potential (EIIP) [21, 22], hydrophobicity [21, 23], solubility [21, 23], or molecular weight [21, 23].

In this paper we introduce CISAPS (complex informational spectrum for the analysis of protein sequences) web server which can be freely accessed to extract features of proteins from their amino acid sequences using the CISA. This is further supported by using an expanded set of amino acid indices (AAI). Application of the CISA in the influenza virus is also presented as a case study in order to show usefulness and robustness of the method developed.

## 2. Methods and Materials

### 2.1. Signal Processing for Protein Sequence Analysis

By using digital signal processing techniques the goal is to extract information that can be related to biological functions of proteins. Various signal processing methods have been used in bioinformatics for analysing protein sequences in recent years; one of the most common methods is the informational spectrum analysis (ISA) [12, 13]. For the ISA method to be implemented for the analysis of protein sequences, discrete Fourier transform (DFT) is applied after each amino acid of the protein sequences is expressed as numerical sequences by using various AAI. A special case of ISA is the resonant recognition model [12, 13, 22] where the EIIP AAI [22] is used to encode alphabetical protein sequences into numerical sequences. ISA reveals that in related protein sequences common peaks appear in the informational spectrum, whereas they do not appear in functionally unrelated sequences, and this is directly related to the biological property of the AAI used. In previous studies, ISA uses DFT to extract parameters using the absolute spectrum. However, DFT that generates complex output (imaginary and real frequency spectra) has been shown to produce complementary information in various fields such as Doppler ultrasound in medicine [14], polar solvation dynamics in the femtosecond evolution [24], time-domain sum-frequency generation spectroscopy using midinfrared pulse shaping [25], hydrophobic oil droplet-water interface for the orientation, and charge of water [26].

To the best of our knowledge, complex signal processing concept has not been explored for the analysis of protein sequences. Therefore, for the first time, this paper is concerned with the development of the complex informational spectrum (CISA) for the analysis of groups of proteins using their sequence information. This study therefore aims at deriving absolute, real, and imaginary spectra from DFT for a given set of proteins. They will then be used to extract characteristic frequency parameters for the group of proteins under study. This piece of information can be used to characterise and classify protein sequences. In order for researchers to apply the method in their own set of proteins without any knowledge of SP or complex SP concept, a freely accessible web server (CISAPS web server) is also developed and presented.

### 2.2. Amino Acid Indices

Protein sequences in the literature are expressed using generally 20 alphabetical characters where each one corresponds to a specific amino acid. To be able to apply signal processing methods protein sequences need to be encoded into numerical sequences. This can be achieved using AAI where each of the 20 amino acids is assigned to a specific numerical value. For the analysis, CISAPS server uses 611 unique AAI to encode protein sequences that represent different biochemical properties of the proteins. A list of all the indices can be retrieved from the CISAPS web server. Of these indices, 528 unique indices were extracted from AA index database [20] after manually removing duplicate entries. The remaining 83 AAI out of 611 used in CISAPS server were retrieved from various literature, the details of which can be found in Supplement 1 in the Supplementary Material available online at http://dx.doi.org/10.1155/2015/909765 and the web server (http://sproteomics.com/cisaps/default/indices).

As AAI originated from different sources from the literature, *z*-score [27] is used to normalise each index using

(1) $$\begin{array}{c} E^{\prime }=\frac{E-\mu \left(E\right)}{\sigma \left(E\right)}, \end{array}$$

where *E*, *μ*, and *σ* correspond to index value, mean value, and standard deviation, respectively, for a particular index.

### 2.3. Preprocessing Protein Sequences

Before applying the complex informational spectrum analysis to the numerical sequences, which have now become signals, preprocessing of these signals is needed, in order for the signal processing methods to be applied in and to extract better results. Recent studies [28] have shown that zero-padding and windowing can enhance the features extracted from proteins sequences. Therefore, both techniques described in this section are applied to the complete protein sequences.

The first technique is windowing where the encoded numerical sequences are multiplied by a precalculated window to reduce spectral leakage. The windowing has been shown to reduce or even eliminate spectral leakage in various applications such as harmonic analysis [29] and phase estimation [30] where frequency analysis and DFT were used. In this case, CISAPS uses Hamming window [31] which can be calculated using (2). The Hamming window is used as it is a widely used and accepted window function [32]:

(2) $$\begin{array}{c} w=0.54-0.46\cos\left(\frac{2\pi \left(N-1\right)}{N-1}\right). \end{array}$$

The second technique used is zero-padding in which a specified number of zero elements are added to the end of each sequence to increase signal length. This technique is essential for CISA as the given protein sequences may not be of the same length. In order to achieve zero-padding, CISAPS server gives two options to the user for analysing a given set of proteins. The first option is to set the resolution directly to the maximum allowed length of any given protein which is 4096 and the second is to set the DFT resolution at the greatest length of the protein sequences given by the user.

### 2.4. Complex Informational Spectrum Analysis

The discrete Fourier transform (DFT) is defined as follows:

(3) $$\begin{array}{c} X\left(n\right)=\overset{N-1}{\underset{m=0}{\sum }}x\left(m\right)e^{-j(2\pi /N)nm}\;n=1,2,\ldots ,N, \end{array}$$

where *x*(*m*) is the *m*th member of the numerical series, *N* is the total number of points in the series, and *X*(*n*) are coefficients of the DFT. As the DFT coefficients consisted of two mirror parts, only the first half of the series (*N*/2) points will be hereafter considered. The following formula determines the maximal frequency in the spectrum:

(4) $$\begin{array}{c} F=\frac{1}{2d}, \end{array}$$

where *F* is the maximal frequency of all the signals (protein sequences) and *d* is the distance between points of the sequence.

If it is assumed that all points of the sequence are equidistant with distance *d* = 1, then the maximum frequency in the spectrum can be found as *F* = 1/2(1) = 0.5. This shows that the frequency range does not depend on the number of points in the sequence but only the resolution of the spectrum. The output of DFT is a complex sequence and can be represented as follows:

(5) $$\begin{array}{c} X\left(n\right)=\left(R\left(n\right)+I\left(n\right)\right),\;n=1,2,\ldots ,\frac{N}{2}, \end{array}$$

where *R*(*n*) and *I*(*n*) are the real and imaginary parts of the sequence, respectively.

The aim of this method is to determine a characteristic frequency peak (CFP) using the informational spectrum for each spectrum (absolute, real, and imaginary) that is expected to correlate with a biological function expressed by a group of protein sequences. To determine such a parameter, it is necessary to find common characteristics of the sequences with the same biological function. The absolute, real, and imaginary informational spectrum can be formulated as follows.

 Absolute spectrum:
  (6) $$\begin{array}{c} S_{a}\left(n\right)=X\left(n\right)X*\left(n\right)={\left|X\left(n\right)\right|}^{2},\;n=1,2,\ldots ,\frac{N}{2}, \end{array}$$

where *S*_*a*_ is the absolute spectrum for a specific protein, *X*(*n*) are the DFT coefficients of the series *x*(*n*), and *X*∗(*n*) are the complex conjugate.

 Real spectrum,
  (7) $$\begin{array}{c} S_{r}\left(n\right)={\left|R(n)\right|}^{2},\;n=1,2,\ldots ,\frac{N}{2}, \end{array}$$

where *S*_*r*_ is the real spectrum for a specific protein and *R*(*n*) are the real parts of DFT coefficients *X*(*n*).

 Imaginary spectrum,
  (8) $$\begin{array}{c} S_{i}\left(n\right)={\left|I(n)\right|}^{2},\;n=1,2,\ldots ,\frac{N}{2}, \end{array}$$

where *S*_*i*_ is the imaginary spectrum for a specific protein and *I*(*n*) are the imaginary parts of DFT coefficients *X*(*n*).

 Complex informational spectrum,
  (9) $$\begin{array}{c} C_{a}=\Pi S_{\left(a\right)}\left(m\right),\;m=1,2,\ldots ,M\\ C_{r}=\Pi S_{\left(r\right)}\left(m\right),\;m=1,2,\ldots ,M\\ C_{i}=\Pi S_{\left(i\right)}\left(m\right),\;m=1,2,\ldots ,M, \end{array}$$

where *C*_*a*_, *C*_*r*_, and *C*_*i*_ are the absolute, real, and imaginary informational spectrum, respectively, and *M* is the number of protein sequences used for a specific class of proteins.

Equation (10) is used to scale absolute, real, and imaginary informational spectrum as

(10) $$\begin{array}{c} V=\frac{\sqrt{\sum_{n=0}^{L}C_{a,r,i}\left(n\right)}}{L}, \end{array}$$

where *L* is the number of points in the absolute (*C*_*a*_), real (*C*_*r*_), and imaginary informational spectrum (*C*_*i*_).

CFP as a result of the CISA can be used to characterise and distinguish them from another group of proteins. However, the following conditions should be fulfilled for the CFP to be related to a biological function.

1. Only one CFP should exist for a group of protein sequences that share the same biological function.
2. For different biological functions the CFP is expected to be different.

In the traditional approach, due to the complex nature of proteins and their functional groups, the use of only the absolute spectrum in the analysis of protein sequences using the informational spectrum analysis is insufficient, as biologically related features to the analysis of protein sequences can be more distinct either in the real or the imaginary spectrum. Some of the applications of ISA and RRM that are already applied in the literature and CISA will also be applicable and will be able to contribute additional information.

## 3. Web Server Access

**Figure 1:**
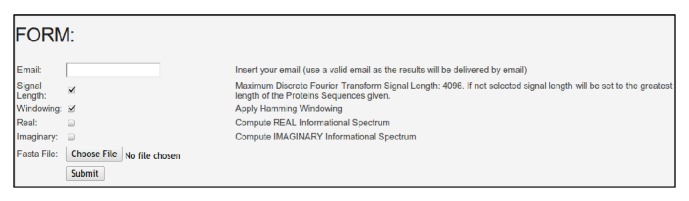
CISAPS web server input form.

The CISAPS web server is available at http://sproteomics.com/cisaps. As seen in Figure 1, the user can input the required information for the analysis using the input form.

The mandatory information required is a valid email and protein sequences saved in FASTA format. The CISAPS web server can process up to 1000 protein sequences per analysis, where the length of any given protein is limited from 8 to 4096. After a successful submission to the CISAPS web server, an email will be sent to the user with a description of the submitted data, including number of proteins, unknown amino acids found in protein sequences, and resolution used for the discrete Fourier transform. After the submission, protein sequences will be processed and an email will be sent to the user with the generated reports of the analysis. The email includes the following:

- a report of the CISA results grouped by CFP,
- a report of the CISA results listed by AAI ID,
- summary report of the occurrences per CFP.

## 4. Case Study: Analysing Influenza Neuraminidase Protein Sequences

During the twentieth century three major influenza A pandemics were recorded which were caused by H1N1, H2N2, and H3N2 viruses in this chronological order. In addition H5N1 and H1N2 viruses are considered as current pandemic threads [33, 34]. Previous studies [17] used influenza A subtypes to analyse the hemagglutinin (HA) gene with the RRM, aiming to identify new therapeutic targets for drug development by better understanding the interaction between the influenza virus and its receptors. For this analysis, the neuraminidase (NA) gene of these five different subtypes of influenza A virus was used, as it is the target for current antiviral drugs, called neuraminidase inhibitors [35]. All the protein sequences were collected from the Influenza Virus Resource database [36].

Influenza A H1N1 subtype virus [37] is a subtype of influenza A virus and the most common cause of influenza in humans. H1N1 first emerged in 1918 and was responsible for Spanish flu that killed 50 to 100 million people worldwide within a year (1918-1919). In 1947 a new H1N1 virus emerged through intrasubtype reassortment while the neuraminidase (NA) gene was preserved, which may have prevented the advancing of a new pandemic. In 1957 H1N1 suddenly became extinct in humans and the reason is still not clear today. One probable explanation is that the development of high immunity to the H1N1 virus in conjunction with the development of immunity to the H2N2 influenza virus led to the extinction of the virus. In 1977 the H1N1 virus reappeared in the former Soviet Union, Hong Kong, and north-eastern China. Genetic analysis of the reemerged H1N1 virus suggests that the strain had been conserved since 1950, and accidentally released from a laboratory facility. In April 2009, a new strain of H1N1 (S-OIV) was identified in the United States. This new strain emerged from reassortment of NA and matrix genes from the Eurasian H1N1 influenza A swine virus and the remaining six gene segments from the H1N2 swine virus. For this subtype, four different groups of proteins (Supplement 2) were retrieved from the Influenza Virus Resource database as follows:

- 27 NA proteins for H1N1 (1933–1946),
- 12 NA proteins for H1N1 (1947–1957),
- 48 NA proteins for H1N1 (1979–1989),
- 200 NA Proteins for H1N1 (2009).

For the influenza A H2N2 subtype, 76 NA proteins were sequenced before the period 1957–1968 as given in the Influenza Virus Resource database. H2N2 influenza viruses that could affect humans appeared in 1957; these were the result of antigenic shift from reassortment between already creating human H1N1 and avian H2N2 viruses [37]. H2N2 viruses possess the HA, NA, and polymerase basic 1 (PB1) gene fragments of an avian H2N2 virus whereas the remaining five gene fragments were originated from human H1N1 virus. H1N1 viruses were displaced by H2N2 viruses that were spreading quickly among humans, causing the Asian flu pandemic (1956–1958) which killed an estimated two million people worldwide [37].

For influenza A H3N2 subtype, 200 NA proteins were retrieved from the Virus Resource database that was sequenced from the period 1968–2000. H3N2 viruses emerged in 1968 by reassortment between circulating human H2N2 and avian H3 viruses [37]. These viruses adapted from H3 avian virus HA and PB1 genes and the six genes, including NA and fragments of the already circulating human H2N2 viruses. H3N2 was responsible for the Hong Kong pandemic (1968-1969) which killed an estimated one million people worldwide.

For Influenza A H1N2 subtype, 27 NA proteins were retrieved from the Virus Resource database that was sequenced from the period 2001–2004. The results of the genetically characterised H1N2 subtype [33] to determine the origin of all eight gene segments showed that all H1N2 isolates were reassortants of classical swine H1N1 and triple reassortant H3N2 viruses. The NA and PB1 genes of the H1N2 isolates were of human origin, while the HA, nucleoprotein (NP), matrix (M), nonstructural (NS), polymerase acidic (PA), and polymerase basic 2 (PB2) genes were of avian or swine origin.

For Influenza A H5N1 subtype, 70 NA proteins were retrieved from the Virus Resource database that was sequenced from the period 2005–2009 in Asia. The H5N1 virus was created by combining various influenza A subtype virus [34]. For H5N1, the PB2, PB1, NP and NS genes originated from Avian H3N8, and the M gene from Avian H7N1. H5N3 has the highest nucleotide similarity to H5N1 for the PA gene, which suggests that it has contributed to the PA and HA gene. Finally Avian H1N1 supplied the NA gene [34].

## 5. Results and Discussion

By submitting each H1N1, H5N1, H2N2, H3N2, and H1N2 NA gene protein file independently in the CISAPS server and using the reports generated for absolute, real, and imaginary informational spectrum, CFPs results were retrieved. All the results obtained, and reports generated can be found in Supplement 3. For the analysis, zero-padding and windowing methods used in signal processing to extract better results were considered. As the influenza A protein sequences have different lengths, maximum DFT resolution as well as windowing was also applied to the signals (protein sequences) in order to reduce spectral leakage as discussed in Section 2.3. A similar CFP between influenza A subtypes would suggest a close relationship between two protein classes for the particular feature that the amino acid index represents. By using minimum and maximum thresholds two sets of AAI were retrieved. The first set represents AAI with identical or closely related CFPs while the second set retrieved, represent amino acids with more distributed CFPs. Two sets of tables are created to illustrate these results; Tables 1, 2, and 3 show AAI that present highly similar cases, where Tables 4, 5, and 6 show AAI that present the most distinct cases according to CFPs. The results produced from CISAPS were ranked according to the similarities and differences based on standard deviation (STD) [38]. The thresholds used for the results presented in this paper are for AAI with identical or closely related CFPs (Tables 1, 2, and 3) smaller than 0.01 and for AAI that present the most distinct results (Tables 4, 5, and 6), larger than 0.2. Further information regarding AAI shown in Tables 1 to 6 can be retrieved from the web server by using the assigned ID number.

After extracting the results from the CISAPS web server, the next step in the analysis is to discover if any of the biological features represented in AAI from Tables 1–6 can be related to previous biological experiments presented in the literature. The following associations were achieved.

- The results indicate that hydrophobicity plays an important role for the neuraminidase gene, as it appears multiple times with different AAI. Identification numbers of these AAI that represent hydrophobicity are 56, 57, 58, 242, and 513. The literature supports [39–41] that the hydrophobic region of the influenza neuraminidase gene plays an important role informing the functionality of the gene [39, 40] and that it is a potential target for new antiviral drugs [39, 40].
- According to the literature protein kinase C (PK-C) which is represented in amino acid 76 appears to play an important role in distinguishing various H5N1 subtypes [42].
- Another protein feature that is utilised from H1N1 subtype mutants [43] is linker propensity, which is represented in AAI 434 and 496.
- Finally, as previous works show, neuraminidase active sites present high polarity [44] which is represented in amino acid index 111.

As the importance of the AAI that represent hydrophobicity, PK-C, and linker propensity to the neuraminidase gene is established, it can be concluded that the rest of the AAI which appear in Tables 1–6 have a higher degree of association than the rest of the AAI in the database. Further biological experiments are required regarding the biological relationship of these indices to the influenza A NA gene. One of the promising results is AAI ID 557 that represent short- and medium-range nonbonded energy [45], which only appears in the imaginary spectrum.

In the literature, when informational spectrum analysis is used [12, 13], only the absolute spectrum is considered. As the results show, only the use of the absolute spectrum to determine how two or more protein classes are related according to CFP is not sufficient. Several AAI do not appear in the absolute spectrum and have significant biological importance to the influenza A NA gene. One example is AAI IDs 111 and 513 (Table 2) that represent polarity and hydrophobicity, respectively. Additionally, AAI IDs for the real informational spectrum are 154, 242, and 427 (Table 2) and for the imaginary informational spectrum are 403, 421, 463, and 557 (Table 3), which do not appear in the absolute spectrum and may also be biologically significant.

## 6. Conclusions

In this paper, a web-based server is developed and presented, named CISAPS, which provides complex informational spectrum analysis for protein sequences. As the results show protein classes that present similarities or differences according to the CFP in specific AAI, it is probable that these classes are related with the protein feature that the specific amino acid represents. Furthermore, the use of only the absolute spectrum in the analysis of protein sequences using the informational spectrum analysis is proven to be insufficient, as biologically related features to the analysis of influenza A subtypes appear individually either in the real or the imaginary spectrum.

CISA approach is a new concept for the protein sequence analysis and can be easily adapted and potentially applied (through its web server) in other areas as described below. 

*Development of New Drugs*. Bioinformatics has become an important component in drug discovery in the recent years, by accelerating this complex, expensive, and time-consuming process. ISA, in combination with the EIIP scale index, can successfully be applied in the bioinformatics model for the discovery and development of new drugs. As the EIIP scale index represents the interaction potential of amino acids, the development time of a new drug can considerably decrease by applying ISA or CISA in the following ways:

1. by extracting key features such as the CFP of compounds that have shown activity against target diseases and comparing them against molecular databases,
2. by using ISA and CISA, the selected compounds can be modified to increase the desired biological activity,
3. additionally potential target areas can be identified by selecting protein or nucleotide sequences domains.

An example of applying ISA in the area of drug discovery can be found in [16] where this technique was applied in development of HIV entry inhibitors and with such further potential applications in HIV/AIDS therapeutic interventions [46], outcome of which suggests targeting the variable region 3 (V3) of the HIV-1 gp120 at the early stage of the infection, which is expected to help potentially develop approaches to designing new HIV/AIDS therapeutic interventions. 

*Identification of Important Protein Sequence's Domains.* In biology, similar or identical nucleotide or protein sequences are called conserved sequences that can occur across different species or presented in different molecules within the same organism. In influenza research area, the identification of such as a conserved domain is essential, especially any receptor binding related domain to the development of influenza inhibitors. By using ISA, the informational [17] and structural [17] features as well as multiple conserved domain [47] of HA with receptor-virus interaction were investigated that relate with receptor-virus interaction. These studies were intended to expand the collection of key regions by discovering multiple domains of H1N1 and H5N1 HA subtype 1 that can alter the receptor binding model. Using the same approach, mutations, F71S, T128S, E302K, and M314L, in the H1N1 HA gene are recognised as necessary for the human interaction. Additionally, positions 94D, 196D, and 274D in the H1N1 HA were marked as important hot spots for mutations. One of these mutations hot spots, D274E, is already identified in H1N1 isolates and its contribution to the human host adaptation is identified. Furthermore, the results in these studies propose that the influenza subtype H1N1 HA gene will persist into mutating, which could further promote the human interaction. These results were extracted using CFP at frequencies 0.055 and 0.295. Another study that uses ISA aims to predict amino acid residues in highly conserved domains of the hormone prolactin (PRL) [19]. In this study, ISA was implemented with the EIIP scale index to extract the CFP of the PRL hormone and to determine which amino acids contribute more to these frequencies, and therefore to the PRL biological function. By using ISA, the highly conserved regions were determined in aminoterminal and C-terminus regions of PRL. As the paper [19] proposes, predictions correspond with experimentally tested residues using site-direct mutagenesis and photoaffinity labelling. 

*Investigation of Protein Sequences Interaction.* Another bioinformatics area in which ISA is applied is the analysis of protein sequence interaction. By using ISA with EIIP index scale's interactions between oncogene, IL-2, and p53 tumor suppressor proteins were analysed [18]. In order to investigate the common interactions of these protein sequences, CFP needs to be determined. As the results of this study have shown, ISA can be effectively used to extract features from protein sequences related to their common biological function. All three interactive protein sequences used share the CFP at frequency 0.0322. This identified feature is a distinguishing feature of oncogene proteins and can be used to characterise promotion of uncontrolled cell growth. Furthermore, anticancerous properties can be identified using CFP features and peptides can be designed to exhibit only these characteristics. As these results [18] show, ISA and CISA can provide a new method to understand information presented in a protein sequence's primary structure. Finally, these results can be used to contribute significantly in the development of new biomaterials by accelerating complex costly and time consuming procedures.

This web-based server enables researchers with little knowledge of signal processing methods to apply and include complex informational spectrum analysis to their work. Furthermore, in the applications discussed above only one amino acid index, commonly EIIP, is used to extract CFP features. CISAPS uses a collection of 611 unique AAI; each one represents a different property to perform the analysis. Moreover, in this paper, various technical issues such as DFT resolution and windowing that may affect the analysis are also addressed.

## Supplementary Material

Supplement 1 provides detailed information about the 611 unique Amino Acid Indices used by the CISAPS server to encode protein sequences to numerical sequences. These Amino Acid Indices represent different biochemical properties of the proteins and are made available at .
Supplement 2 provides the protein sequences of influenza Neuraminidase gene used as the case study presented in the paper. Eight different groups of the proteins are included as follows: (i) 27 NA proteins for H1N1 (1933–1946) (ii) 12 NA proteins for H1N1 (1947–1957) (iii) 48 NA proteins for H1N1 (1979–1989) (iv) 200 NA Proteins for H1N1 (2009) (v) 76 NA Proteins for H2N2 (1957–1968) (vi) 200 NA Proteins for H3N2 (1968–2000) (vii) 27 NA Proteins for H1N2 (2001–2004) (viii) 70 NA Proteins for H5N1 (2005–2009).
Supplement 3 provides the results produced by CISAPS Server as an outcome of the analysis of the protein sequences included in Supplement 2. The results include reports generated for absolute, real, and imaginary informational spectrum characteristic frequency peaks.

## Figures and Tables

**Table 1:** Characteristic frequency peaks for absolute informational spectrum for similar cases.

AAI ID	H1N1 1933	H1N1 1947	H1N1 1979	H1N1 2009	H5N1	H1N2	H2N2	H3N2	STD
56	0.142	0.1418	0.1418	0.1276	0.1423	0.122	0.1218	0.122	0.0101
57	0.1276	0.1276	0.1276	0.1276	0.1274	0.122	0.1218	0.122	0.0029
58	0.142	0.142	0.142	0.1276	0.1425	0.122	0.122	0.122	0.0102
84	0.082	0.0817	0.082	0.0815	0.0817	0.081	0.0813	0.081	0.0004
113	0.1208	0.1208	0.1208	0.1205	0.1208	0.122	0.122	0.122	0.0007
126	0.082	0.0817	0.0817	0.0817	0.0815	0.081	0.0813	0.081	0.0004
173	0.082	0.0817	0.082	0.0817	0.0815	0.0808	0.0813	0.081	0.0004
333	0.0817	0.0815	0.0817	0.0815	0.0813	0.0805	0.081	0.0808	0.0004
369	0.082	0.0817	0.082	0.0817	0.0815	0.081	0.0813	0.0813	0.0004
416	0.0815	0.0815	0.0815	0.0813	0.081	0.0803	0.0808	0.0805	0.0005
436	0.1271	0.1271	0.1271	0.1271	0.1269	0.122	0.122	0.122	0.0026
544	0.1274	0.1274	0.1274	0.1274	0.1271	0.1218	0.1218	0.1218	0.0029
589	0.0822	0.082	0.082	0.0817	0.082	0.0815	0.0815	0.0815	0.0003

**Table 2:** Characteristic frequency peaks for real informational spectrum for similar cases.

AAI ID	H1N1 1933	H1N1 1947	H1N1 1979	H1N1 2009	H5N1	H1N2	H2N2	H3N2	STD
56	0.1269	0.1271	0.1423	0.1271	0.1418	0.1218	0.1218	0.1218	0.0085
57	0.1269	0.1271	0.1271	0.1271	0.1274	0.1218	0.1218	0.1218	0.0028
58	0.1269	0.1425	0.1423	0.1425	0.1418	0.1218	0.1218	0.1218	0.0104
84	0.0827	0.0813	0.0813	0.0813	0.0817	0.0813	0.0813	0.0813	0.0005
111	0.1269	0.1271	0.1271	0.1271	0.1274	0.1218	0.1218	0.122	0.0027
113	0.121	0.1213	0.1213	0.1213	0.121	0.1218	0.1218	0.1218	0.0003
126	0.0827	0.0813	0.0813	0.0813	0.0817	0.0813	0.0815	0.0815	0.0005
173	0.0827	0.0813	0.0813	0.0813	0.0817	0.0813	0.0815	0.0815	0.0005
242	0.1269	0.1271	0.1271	0.1271	0.1271	0.1218	0.1218	0.1218	0.0027
333	0.0827	0.081	0.0813	0.0813	0.0817	0.0813	0.0813	0.0813	0.0005
369	0.0827	0.0813	0.0813	0.0813	0.0817	0.0813	0.0815	0.0815	0.0005
416	0.0825	0.081	0.0813	0.0813	0.0817	0.0813	0.0813	0.0813	0.0005
436	0.1269	0.1271	0.1271	0.1271	0.1274	0.1215	0.1215	0.1215	0.0029
513	0.1269	0.1271	0.1271	0.1271	0.1274	0.1218	0.1218	0.1218	0.0028
544	0.1269	0.1271	0.1271	0.1271	0.1274	0.1218	0.1218	0.1218	0.0028
589	0.0827	0.0813	0.0813	0.0813	0.082	0.0815	0.0815	0.0815	0.0005

**Table 3:** Characteristic frequency peaks for imaginary informational spectrum for similar cases.

AAI ID	H1N1 1933	H1N1 1947	H1N1 1979	H1N1 2009	H5N1	H1N2	H2N2	H3N2	STD
56	0.1279	0.1415	0.1415	0.1281	0.1428	0.1227	0.1225	0.1227	0.0092
57	0.1281	0.1281	0.1281	0.1281	0.1428	0.1227	0.1227	0.1225	0.0066
58	0.1281	0.1415	0.1415	0.1281	0.1428	0.1227	0.1225	0.1227	0.0092
84	0.0817	0.082	0.0822	0.0822	0.0827	0.0803	0.0803	0.0803	0.0010
113	0.1201	0.1203	0.1203	0.1203	0.1201	0.1227	0.1227	0.1225	0.0013
126	0.0817	0.0822	0.0822	0.0822	0.0808	0.0805	0.0805	0.0805	0.0008
173	0.0817	0.0822	0.0825	0.0822	0.0808	0.0803	0.0805	0.0805	0.0009
333	0.0817	0.0822	0.0822	0.0822	0.0808	0.0803	0.0805	0.0803	0.0009
369	0.0817	0.0822	0.0822	0.0822	0.0808	0.0803	0.0805	0.0803	0.0009
403	0.0378	0.0698	0.0698	0.0698	0.0713	0.0698	0.0698	0.0698	0.0114
416	0.0815	0.082	0.082	0.082	0.0805	0.0803	0.0803	0.0803	0.0008
436	0.1281	0.1281	0.1281	0.1281	0.1262	0.1225	0.1225	0.1225	0.0028
463	0.4846	0.4832	0.4832	0.4832	0.4841	0.4851	0.4849	0.4851	0.0009
544	0.1281	0.1281	0.1281	0.1281	0.1428	0.1227	0.1227	0.1225	0.0066
589	0.0817	0.0822	0.0825	0.0822	0.083	0.0805	0.0825	0.0825	0.0008

**Table 4:** Characteristic frequency peaks for absolute informational spectrum for dissimilar cases.

AAI ID	H1N1 1933	H1N1 1947	H1N1 1979	H1N1 2009	H5N1	H1N2	H2N2	H3N2	STD
73	0.4885	0.4712	0.471	0.471	0.4707	0.02	0.02	0.0203	0.2352
81	0.4837	0.4839	0.0251	0.0576	0.0588	0.4861	0.4861	0.4861	0.2269
110	0.4605	0.4605	0.4607	0.0203	0.02	0.0205	0.0203	0.0205	0.2278
285	0.0586	0.0586	0.4341	0.4344	0.4346	0.0207	0.4363	0.0205	0.2118
359	0.4888	0.4888	0.4885	0.0188	0.4885	0.0215	0.458	0.0215	0.2393
373	0.4893	0.4898	0.49	0.4893	0.489	0.0769	0.0761	0.0764	0.2138
375	0.4297	0.4292	0.4283	0.4305	0.43	0.0381	0.0378	0.0378	0.2027
496	0.4463	0.4463	0.4466	0.3902	0.0234	0.0683	0.0686	0.0686	0.2019
536	0.4602	0.4602	0.4605	0.4602	0.4602	0.0203	0.0207	0.0205	0.2276
574	0.0395	0.0395	0.0395	0.0393	0.3502	0.4863	0.4858	0.4861	0.2250
588	0.0576	0.0573	0.0573	0.0576	0.0583	0.4863	0.4861	0.4863	0.2218

**Table 5:** Characteristic frequency peaks for real informational spectrum for dissimilar cases.

AAI ID	H1N1 1933	H1N1 1947	H1N1 1979	H1N1 2009	H5N1	H1N2	H2N2	H3N2	STD
73	0.4641	0.4702	0.4705	0.4705	0.4824	0.0195	0.0195	0.0195	0.2340
76	0.4702	0.4841	0.4841	0.0859	0.0727	0.409	0.021	0.021	0.2224
81	0.0246	0.4841	0.0249	0.1796	0.0591	0.4858	0.4858	0.4858	0.2261
110	0.4602	0.4605	0.4605	0.0193	0.021	0.4566	0.0195	0.4566	0.2272
154	0.0581	0.4841	0.0561	0.0561	0.0591	0.3773	0.4858	0.4858	0.2172
343	0.4902	0.1083	0.1083	0.0815	0.49	0.1088	0.4339	0.4339	0.1939
359	0.4883	0.4888	0.4885	0.0193	0.0188	0.0215	0.4585	0.0215	0.2465
373	0.491	0.4888	0.489	0.4888	0.4905	0.0781	0.0761	0.0761	0.2137
375	0.43	0.428	0.4278	0.43	0.4305	0.0371	0.0371	0.0371	0.2030
427	0.0224	0.0227	0.0227	0.3895	0.0224	0.4366	0.4366	0.3199	0.2027
536	0.4605	0.4605	0.4607	0.0212	0.4595	0.0193	0.0212	0.0212	0.2350
574	0.0403	0.04	0.04	0.0398	0.039	0.4863	0.4861	0.4861	0.2310
588	0.0581	0.0581	0.0581	0.0581	0.0588	0.4861	0.4858	0.4858	0.2213

**Table 6:** Characteristic frequency peaks for imaginary informational spectrum for dissimilar cases.

AAI ID	H1N1 1933	H1N1 1947	H1N1 1979	H1N1 2009	H5N1	H1N2	H2N2	H3N2	STD
73	0.4632	0.4714	0.4714	0.4714	0.4837	0.0205	0.0203	0.0205	0.2339
76	0.0722	0.4832	0.4832	0.0717	0.4839	0.408	0.0754	0.0754	0.2104
81	0.0259	0.4834	0.0261	0.0571	0.0578	0.4871	0.4868	0.4868	0.2378
285	0.4334	0.1096	0.4336	0.4336	0.4346	0.0203	0.4356	0.0205	0.2007
359	0.4893	0.4898	0.4898	0.0183	0.4888	0.0224	0.0205	0.0205	0.2507
375	0.4307	0.429	0.429	0.4309	0.4295	0.0381	0.0381	0.0381	0.2027
343	0.4893	0.1074	0.1074	0.0825	0.489	0.0637	0.4351	0.4348	0.2003
421	0.0232	0.0237	0.0237	0.3902	0.0234	0.4358	0.4356	0.4356	0.2148
434	0.4653	0.0237	0.0237	0.4653	0.0234	0.4356	0.4356	0.4356	0.2197
536	0.4649	0.185	0.4597	0.4597	0.4605	0.0203	0.0203	0.0203	0.2204
557	0.0569	0.02	0.02	0.0571	0.0195	0.4085	0.4361	0.4361	0.2037
574	0.0393	0.039	0.039	0.0388	0.0403	0.4873	0.4851	0.4854	0.2312
588	0.0569	0.4029	0.0571	0.0571	0.0578	0.4868	0.4868	0.4868	0.2201
